# Examining Students’ Online Learning Satisfaction during COVID-19 to Inform Post-Pandemic Program Planning

**DOI:** 10.3390/nursrep13010031

**Published:** 2023-02-23

**Authors:** Mohammad Alasagheirin, Jeanette M. Olsen, Dalete Mota, Meg Lagunas, Benjamin D. Bogle

**Affiliations:** Department of Nursing, University of Wisconsin–Eau Claire, Eau Claire, WI 54702, USA

**Keywords:** online learning, nursing education, satisfaction, stress, resilience

## Abstract

Background: Student satisfaction is one of five pillars of quality online learning and is associated with academic achievement. This study aimed to examine nursing students’ satisfaction with online learning during COVID-19, their desire to continue online classes, and associated factors. Methods: A cross-sectional survey was completed by 125 nursing students from a public university. The students’ satisfaction was measured with the Student’s Satisfaction Towards Online Learning Questionnaire. Demographics, stress, and resilience were also measured. Data were analyzed using descriptive statistics and multiple logistic regression. Results: Fewer than half (41.8%) of students were satisfied with online learning. Just over half (51.2%) did not want to continue with online classes. The strongest predictor of satisfaction was course management and coordination. The strongest predictor for a preference to continue with online classes was the instructor’s characteristics. Conclusions: Considering the trend of providing more online education to nursing students, instructors should be prepared for excellence in online course management and coordination as the instructors have a fundamental role in students’ satisfaction with online learning. Further examination of nursing students’ satisfaction with online learning during the pandemic may yield valuable insights for post-pandemic program planning.

## 1. Introduction

Online learning is not new in nursing. As a mode of distance education, online learning, characterized by having at least 80% of the delivery by computer and internet, is now common in higher education [1]. Even before the COVID-19 pandemic, interest in online learning was growing and approximately one-third of United States (US) college students were enrolled in at least one online course [2]. Most college students (63%) who chose to study online did so because it fit their work and personal responsibilities [3]. Likewise, some nursing students elected to enroll in an online program or courses. Unfortunately, the pandemic eliminated this choice. All nursing students were required to become online learners, regardless of learning preferences, strengths, or attributes. 

Despite the challenges imposed by the COVID-19 pandemic, online learning has benefits. A landmark meta-analysis conducted by the US Department of Education [4] found that student learning outcomes following distance education were, at minimum, equivalent to those of traditional face-to-face (F2F) courses. McCutcheon et al. [5] examined whether clinical skills teaching was enhanced through online or blended learning. This systematic review (n = 19 studies) concluded that online learning of clinical skills is as effective as traditional methods. Additional reported benefits of distance education include increased access to courses, more flexible learning options, improved course consistency in programs with multiple sites, and the potential to ease nursing faculty shortages [6,7]. Throughout the current COVID 19 pandemic, online learning provided a platform for students to continue their programs of study and protected the health and well-being of both educators and students. 

Many colleges anticipate post-pandemic growth in online learning that surpasses pre-pandemic rates [8]. Examining nursing students’ satisfaction with online learning during the pandemic may yield valuable insights for post-pandemic program planning. Student satisfaction is one of the five pillars of quality online learning [9]. It has been associated with higher learning engagement [10] and academic achievement [11]. Results from studies on nursing students’ satisfaction with online learning during the pandemic have been inconsistent with some authors reporting most students were satisfied [12,13] and others reporting most preferred F2F learning [14,15]. Multiple associated factors have been suggested including learner characteristics [12,13], course management [11,13], adequate technology [16], resilience [17], and program level [14,16]. For example, Alqahtani et al. [12] surveyed 139 nursing students in Saudi Arabia to determine factors associated with satisfaction with e-learning. Results indicated the students were satisfied and presented high readiness for e-learning; the two scores were correlated (r = 0.602; *p* < 0.001). The authors also reported that previous experience with and readiness for e-learning explained 40.2% of the variance in overall satisfaction (*p* < 0.001). Sharma et al. [13] surveyed 434 undergraduate and graduate students from a medical college in Nepal, 28.4% of whom were nursing students, and found 53.5% were satisfied with online learning during the pandemic. Multivariate analysis indicated female gender, WiFi internet for learning, and learners’ dimension score (a measure of learner characteristics) were significant predictors of satisfaction. Additionally, 89.8% of students reported they would like to continue online classes during the pandemic.

In contrast to the findings reported by Alqahtani et al. [12] and Sharma et al. [13], results from several studies indicated that most students were not satisfied with online learning during the pandemic. Dutta et al. [18] examined satisfaction with pre-clinical and clinical distance education during the pandemic among medical (n = 919) and nursing (n = 149) students in India. The authors found that 38% were satisfied or very satisfied with online learning. Although the level of satisfaction did not differ significantly between medical and nursing students (*p* = 0.192), first-year students were significantly more dissatisfied than senior students (*p* = 0.005). Students were most satisfied (78%) with faculty supportiveness and responsiveness and least satisfied (46%) with communication and discussion with peers. Similarly, Li et al. [19] explored factors influencing satisfaction with online learning among medical (n = 207) and nursing (n = 23) students in China and reported over 60% of the students were dissatisfied. Dissatisfaction was higher among students in their clinical years (73%) compared to those in the basic year (59%) (*p* < 0.05). Li et al. [19] found forty factors significantly influenced students’ satisfaction with online learning, the most influential being: well-accomplished course assignments, adequate internet access, adequate support from the university, self-discipline, and adequate use of course resources. Langegård et al. [15] conducted a mixed methods study to analyze nursing students’ experience transitioning to distance learning. Findings from focus group interviews (n = 9) revealed three themes: didactic aspects of navigating the digital learning environment; students’ physical and psychological study environment; and students’ motivation, discipline, and responsibility. Quantitative results (n = 96) indicated about two-thirds of students preferred traditional, on-campus learning. There was a significant deterioration in all online learning domains: 63% in course content, 66% in student-teacher communication, 68% in practical information about the course, 63% in motivation, 58% in study discipline, and 40% in responsibility. More recently, Bowser et al. [14] examined satisfaction with remote and F2F learning among undergraduate and doctoral nursing students (N = 522) attending a research-intensive university in the Eastern US. They found the overall mean satisfaction was higher for F2F learning (*p* < 0.001) but varied across programs, with BSN students being less satisfied with remote learning than DNP and PhD students. The authors also found results differed by BSN class level (*p* = 0.004); first year and senior students were more dissatisfied with remote learning compared to sophomore and junior students. 

The unexpected transition to online learning, combined with COVID-19 uncertainties regarding infection and disrupted social interactions, has been associated with elevated stress and anxiety levels among college students [20,21]. Wallace et al. [17] conducted a qualitative study exploring the experience of prelicensure nursing students (N = 11) transitioning to remote learning. Four themes were identified: technological challenges (communication; connectivity; faculty aptitude), academic relationship changes (isolated from peers; deterioration of student-faculty relationships), role stress and strain (more family responsibilities; challenges learning at home), and resilience. Fitzgerald and Konrad [22] examined stress and anxiety experienced by first-semester baccalaureate nursing students (N = 50) during the transition to online learning in the first few months of the pandemic. The authors found that 90% of the students experienced difficulty concentrating, 84% felt anxious or overwhelmed, 70% were concerned about their own health, and 62% had concerns about handling the academic workload. Nursing students also faced disruption and uncertainties regarding clinical training requirements, especially in the third and fourth years [23]. Finally, Kim and Park [24] found anxiety from COVID-19 moderated the relationship between online learning satisfaction and outcomes. This is notable since the relationship between mental health factors and nursing students’ satisfaction with online learning has been minimally explored [24]. 

As described above, current literature on nursing students’ satisfaction with online learning during the COVID-19 pandemic demonstrates international interest with inconsistent findings; however, dissatisfaction was prevalent. Notably, most of these studies occurred early in the pandemic capturing students’ perspectives on the emergent implementation of online learning. However, the pandemic has persisted for over two years. More research is needed to clarify nursing students’ satisfaction with online learning during the pandemic. This knowledge may help nurse educators improve the effectiveness of online instruction and may help nursing programs discern which courses and programs should continue in an online learning setting. Thus, this study aimed to examine nursing students’ satisfaction with online learning during COVID-19, their desire to continue online classes, and associated factors. The following research questions were addressed:

1. What is the nursing students’ level of satisfaction with online learning during the COVID-19 pandemic?
2. What are nursing students’ levels of stress and resilience?
3. What factors are associated with students’ satisfaction with online learning and their desire to continue online classes?

## 2. Materials and Methods

### 2.1. Design, Setting, and Participants 

This cross-sectional study took place in a mid-sized public university in the Midwest United States with traditional Bachelor of Science in Nursing (TBSN), Bachelor of Science in Nursing Completion (BSNC), Master of Science in Nursing (MSN), and Doctor of Nursing Practice (DNP) programs. Prior to the pandemic, nursing courses were predominantly delivered F2F. A mandatory shift to online learning occurred in all courses in March 2020. Beginning in the Fall of 2020, students gradually returned to onsite clinical, but didactic courses remained online until Spring 2022. All nursing students that were enrolled in courses between May 2020 and November 2021 were eligible to participate in this study. There were no exclusion criteria. An email announcing the study with a link to an electronic survey was sent to all students in the Fall of 2021. Three reminders were sent by email to improve the response rate, and the survey was available for a total of eight weeks. 

### 2.2. Study Procedures and Measurement Tools 

Study data were collected using Qualtrics survey software (Qualtrics XM, September 2021, Provo, UT, USA). Consent to participate was obtained electronically. Demographic measures included age, gender, race, and nursing program. 

Satisfaction toward online learning during the COVID-19 pandemic was measured using the Students’ Satisfaction Towards Online Learning Questionnaire (SSTOLQ) [13]. The SSTOLQ consists of 31 items rated on a five-point Likert scale (1 = strongly disagree; 3 = neutral; 5 = strongly agree) and one yes/no item. The first 29 items address four domains of satisfaction: learner’s dimension (nine items that measure learners’ attitude and readiness toward online learning), instructor’s characteristics (nine items that measure instructors’ interactions with learners and content delivery), technological characteristics (seven items that measure the effectiveness of electronic media), and course management and coordination (four items that measure content, access to learning materials, and instructions). A score for each domain is calculated by averaging responses to items with the domain. The final questions ask about students’ overall satisfaction toward and helpfulness of online classes (Likert scale) and if students want to continue online classes (yes/no). Sharma et al. [13] reported high instrument validity and reliability. Internal consistency in the present study was high (α = 0.92). 

The stress level was measured using the stress subscale within the Depression Anxiety Stress Scale-21 (DASS-21), which consists of seven items [25]. Each item is rated on a four-point Likert scale (0 = did not apply to me at all; 4 = applied to me very much or most of the time). Responses are summed and categorized as normal, mild, moderate, severe, or extremely severe stress. DASS-21 reliability and validity were reported as high [25], and internal consistency in this study was high (α = 0.89). 

Resilience was measured with the Brief Resilience Scale (BRS) [26]. The BRS consists of six questions rated on a five-point Likert scale (1 = strongly disagree; 3 = neutral; 5 = strongly agree). Responses are summed and categorized as very low, low, moderate, high, and very high [26]. For this study, the low and very low categories were combined, as were the high and very high categories, resulting in three groups (low, moderate, and high). Internal consistency also was high for this scale (α = 0.86).

### 2.3. Data Analysis 

Data were analyzed using the IBM Statistical Package for Social Sciences (SPSS) version 27. Means and standard deviations (SDs) were calculated for continuous measures (age; SSTOLQ domain scores). Frequencies and percentages were calculated for categorical measures (gender, race, and nursing program; satisfaction with and desire to continue online classes; stress and resilience). Relationships between variables were described using Spearman’s and Pearson’s rank correlation coefficients, as appropriate. Multivariate logistics regression models were constructed to identify predictors of students’ overall satisfaction with online courses and their preference to continue with online learning. Statistical significance was set at *p* < 0.05. 

### 2.4. Ethical Considerations 

The University’s Institutional Review Board approved the study following the US Federal Policy for the Protection of Human Subjects (Process: 202120392). When participants filled out and submitted the survey, informed consent was considered implied. 

## 3. Results

### 3.1. Participants’ Characteristics

A total of 823 nursing students across all programs were eligible to participate in the study; 148 responded, and 125 completed the survey and were included in the final sample (response rate of 15.2%). The mean age was 24.0 years (range: 18–51 years). Most were female (89.6 %; n = 112) and most were white non-Hispanic (91.2%; n = 114). The highest proportion of the students was from the TBSN program (47.2%; n = 59), followed by pre-nursing (27.2%; n = 34), totaling 93 participants enrolled in the TBSN program. Six (4.8%) were in the BSNC program and 26 (20.8%) were in the DNP program. No students from the MSN program answered the survey. Since the number of BSNC responses was low and most students in the program were non-traditional students working as registered nurses (RNs) during the pandemic, BSNC and graduate programs students (20.8%; n = 26) were combined to form one post-licensure group for analysis by program. 

### 3.2. Satisfaction, Stress, and Resilience towards Online Classes

When asked about overall satisfaction towards online classes, 36.8% (n = 46) responded as satisfied or strongly satisfied. Over half (51.2 %; n = 64) reported they did not want to continue with online classes. Overall satisfaction towards online classes and the desire to continue with online classes were compared across programs. Post-licensure students had the highest satisfaction rate (64.3% satisfied or strongly satisfied) and the highest preference to continue online classes (67%). In contrast, 39% of pre-nursing students and 33% of TBSN students reported satisfaction or strong satisfaction with online classes, and the desire to continue online classes was expressed by 28% of pre-nursing and 35% of TBSN students. The SSTOLQ domain scores were positive with overall means of 29.17 (SD = 6.88) for learner’s dimension (neutral = 27), 31.49 (SD = 6.7) for instructor’s characteristics (neutral = 27), 25.99 (SD = 2.83) for technological characteristics (neutral = 21), and 14.57 (SD = 2.92) for course management and coordination (neutral = 12). Statistically significant differences by program were found for the learner’s dimension (*p* = 0.019) and course management and coordination domains (*p* = 0.009) with post-licensure students found to have higher satisfaction than pre-nursing and TBSN students for both. 

Most students (82.2%; n = 88) reported severe or extremely severe stress. This included 90.7% (n = 49) of TBSN students, 79.2% (n = 19) of pre-nursing students, and 69% (n =20) of post-licensure students (*p* < 0.001). Overall, only 17.9% (n = 19) of students showed high resilience, whereas 36.8% (n = 39) showed low resilience. There were significant differences across programs with post-licensure students having the highest resilience (35.7% with high resilience) compared to 17.4% pre-nursing and 9.1% TBSN students (*p* = 0.041). 

### 3.3. Predictors of Satisfaction towards Online Classes

For the multiple regression modeling to predict students’ overall satisfaction towards online learning, those who indicated they were neutral (n = 22) regarding satisfaction with online classes were excluded from the analysis and the satisfaction variable was dichotomized as dissatisfied (dissatisfied and strongly dissatisfied responses) or satisfied (satisfied or strongly satisfied responses). Direct logistic regression was performed with a set of predictor variables: age, gender, program, stress, resilience, and the four SSTOLQ domains. All variable measures were entered in the model as described in the Methods section except the resilience variable, which was categorized as low or high. The model was statistically significant (X2 (9, N = 81) = 83.662, *p* < 0.001), indicating that it was able to distinguish between those who reported satisfaction with online learning and those who reported dissatisfaction (Table 1). The model correctly classified 95.1% of the cases. Four of the independent variables made a unique, statistically significant contribution to the model: learner’s dimension, course management and coordination, instructor’s characteristics, and stress. The strongest predictor of student satisfaction with online learning was course management and coordination with an odds ratio of 3.867 (*p* = 0.007, CI = 1.455, 10.278). This indicates the odds were 3.867 times greater that students who had a higher satisfaction with course management and coordination would be satisfied with online learning after controlling for all other variables in the model. Students who had higher learner’s dimension scores were 2.176 (*p* = 0.001, CI = 1.35, 3.505) times more likely to report overall satisfaction with online learning after controlling for all other variables in the model. 

### 3.4. Predictors of Preference to Continue with Online Classes

A second multiple regression model was tested to predict students’ preference to continue with online classes. The model was statistically significant (X2 (9, N = 101) = 67.377, *p* < 0.001), indicating that it was able to distinguish between students who reported a preference to continue with online classes and those who did not (Table 2). The model correctly classified 83.2% of cases. Three independent variables made a unique, statistically significant contribution to the model: learner’s dimension, course management and coordination, and instructor’s characteristics. The strongest predictor of students’ preference to continue with online classes was instructor’s characteristics with an odds ratio of 1.33 (*p* = 0.002, CI = 1.114, 1.594). This indicates the odds were 1.33 times greater that students who had higher satisfaction with the instructor’s characteristics would prefer to continue with online classes after controlling for all other variables in the model.

## 4. Discussion

Findings from this study indicated fewer than half of participating students were satisfied with online learning during the COVID-19 pandemic and just over half did not want to continue with online classes. However, when results were examined by program, most post-licensure students from the BSNC and graduate programs reported both satisfaction and a desire to continue online learning. Bowser et al. [14] reported similar results from their study of nursing students’ satisfaction with remote and F2F learning one year post COVID restrictions. They found traditional BSN students had consistently higher satisfaction with F2F learning compared to students from accelerated BSN and graduate programs. The higher satisfaction with and desire to continue online classes among BSNC and graduate students in the present study is notable since some BSNC and most graduate nursing courses in this university were delivered in a hybrid format that required multiple and sometimes weekly F2F classes prior to the pandemic when students enrolled in these programs. Additionally, the difference between TBSN and BSNC graduate student satisfaction with online classes was expected. This expected difference is likely due to the natural differences between the life experiences of the students. Graduate students often have family and work obligations motivating them to appreciate and seek the increased flexibility of online classes.

Our findings indicate the shift to online learning during the pandemic may have altered perceptions among this group of non-traditional students. More research is needed to discern precisely how perspectives may have changed. Even so, these findings have implications for program-level planning. Post-licensure and graduate programs that have traditionally been delivered predominantly through the F2F format may want to examine students’ perspectives and preferences as we emerge from the pandemic and use findings to determine if a larger portion of the program should be delivered online. Additional data sources such as student learning outcomes, course evaluations, program exit surveys, and available resources should also be considered when making this decision.

This study indicated that most nursing students were very stressed, and stress levels were negatively associated with satisfaction in online learning. Students’ stress levels, measured 18 months after the pandemic started, were higher than stress levels measured with the same tool in prior studies conducted in the initial months of the COVID-19 pandemic [27,28]. Nursing students consistently report higher stress levels than other college students [29], however, the higher stress levels in the present study may reflect the cumulative and prolonged effects of the transition to online learning, meeting academic expectations, limited socialization, and fear of contagion. Educators can play a vital role in decreasing students’ stress by creating clear academic expectations, avoiding excessive course assignments and workloads, creating a collaborative online-class environment, and adopting positive coping strategies such as counseling and meditation. 

Resilience can mitigate the effects of stress and support well-being and success in nursing. Our results indicated resilience among nursing students was low; however, it did not significantly predict satisfaction with online learning or the desire to continue with online classes. These findings are surprising since an integrative review of 17 studies indicated resilience was one of five key elements contributing to satisfaction in the learning journey [30]. The difference in our findings may be due to the demographics of our sample. For example, post-licensure students in the current study reported higher resilience than pre-nursing and TBSN students, which connects with the literature. Other researchers have described similar results, noting resilience is associated with levels in the nursing program and age [31]. Emerging evidence on resilience highlights the interaction between multifaceted factors and suggests fortitude and the grit to persevere should be examined in future studies [32,33]. 

In the present study, the strongest predictor of satisfaction with online learning was course management and coordination. This finding is consistent with earlier studies in which robust course assignments and ease of navigation in the digital environment were identified as factors associated with online learning satisfaction [15]. Based on these results, nurse educators who teach online should thoughtfully strengthen course management and coordination elements in their courses. Examples include increasing virtual accessibility of content, clarity of instructions for assignments and participation, and learner access to instructors. Access to university resources and experts should be available to help new and seasoned faculty enhance their knowledge and skills in online course design and management strategies. This content should also be included in graduate programs preparing nurse educators. Future studies are needed to determine if implementing strategies to improve course management and coordination will increase student satisfaction with online learning and learning outcomes. 

Findings from our regression models suggest that factors associated with online learning satisfaction and those associated with the desire to continue online classes differ. Notably, in our study, the multiple logistic regression modeling indicated the strongest predictors of the desire to continue online classes were satisfaction with the instructor’s characteristics and technological characteristics. Thus, the results demonstrated that the relationship between instructor and students is a key component for satisfaction, which in turn increases their learning engagement and their academic achievement potential. As we emerge from the pandemic, nursing programs should explore these topics discreetly. First, programs should determine if online learning is preferred by the learner population(s) and would support students’ attainment of learning outcomes. Then, in courses and programs for which online learning is continued, programs should strengthen factors associated with satisfaction. Finally, although stress and resilience were not among the strongest predictors in either model, the high stress and low resilience levels reported in this study need further exploration and intervention. 

This study has limitations. Results were derived from a small, homogeneous sample in one university; therefore, findings may not be generalizable to all nursing programs. Furthermore, the authors acknowledge the low response rate (15.2%) which may be explained by the high demands on nursing students and the timing of the data collection within the academic semester. Future recommendations would be to time the data collection in the middle of the semester and leave the survey open for a longer time. Data were self-reported using an online survey administered during the COVID-19 pandemic which may have influenced results. Additionally, a cross-sectional design was used, limiting the ability to infer a cause-effect relationship between study variables. 

## 5. Conclusions

Results from this study of nursing students in multiple programs within one university revealed factors associated with online learning satisfaction and program-level differences regarding the desire to continue online classes that can be used for post-pandemic program planning. Nursing students’ level of satisfaction with online learning was mixed. This study revealed that post-licensure students’ preference to continue online classes was significantly higher than pre-nursing and TBSN students. Further, factors associated with satisfaction with online learning and factors associated with a desire to continue online classes were different. The strongest predictor of overall satisfaction was satisfaction with course management and coordination. In contrast, the strongest predictors of the desire to continue online classes were the instructor’s characteristics and technological characteristics. Resuming pre-pandemic operations without pausing to examine how learners may have changed and how satisfaction with online learning can be improved eliminates a valuable opportunity for programs to capitalize on what can be learned following this unprecedented experience. The COVID-19 pandemic transformed nursing education. Therefore, nurse educator programs should integrate online course coordination and management competencies into program curricula. Online nursing courses should be staffed to support adequate faculty accessibility for all students, and resources to help both new and seasoned faculty enhance knowledge and skills in online course design and management strategies should be provided.

## Figures and Tables

**Table 1:** Logistic Regression of Factors Related to Satisfaction towards Online Learning (N = 81).

	B (SE)	*p*	95% CI for Odds Ratio		
			Lower	Odds Ratio	Upper
Age	0.132 (0.297)	0.658	0.637	1.141	2.042
Gender	0.944 (1.995)	0.636	0.051	2.570	128.348
Nursing program level					
3 (ref; Post-licensure)		0.824			
1 (Pre-nursing)	0.292 (1.829)	0.873	0.037	1.339	48.261
2 (TBSN)	−1.155 (3.508)	0.742	0.000	0.315	304.854
Stress level	−0.315 (0.149)	0.034 *	0.545	0.730	0.977
Resilience	3.298 (2.534)	0.193	0.189	27.052	3881.278
Learner’s dimension domain	0.777 (.243)	0.001 *	1.350	2.176	3.505
Instructor’s characteristics domain	−0.429 (0.165)	0.009 *	0.472	0.651	0.899
Technological characteristics domain	−0.334 (0.261)	0.200	0.429	0.716	1.193
Course management and coordination domain	1.353 (0.499)	0.007 *	1.455	3.867	10.278
Constant	−20.923 (13.131)	0.111		0.000	

* *p* < 0.05. Model X2 (9, N = 81) = 83.662, *p* < 0.001.

**Table 2:** Logistic Regression of Factors Related to Preference to Continue Online Classes (N = 101).

	B (SE)	*p*	95% CI for Odds Ratio		
			Lower	Odds Ratio	Upper
Age	−0.370 (0.092)	0.666	0.811	0.963	1.144
Gender	−0.635 (1.261)	0.614	0.045	0.530	6.268
Nursing program level					
3 (ref; post-licensure)		0.185			
1 (Pre-nursing)	−1.799 (0.981)	0.067	0.024	0.165	1.132
2 (TBSN)	−1.559 (1.480)	0.292	0.012	0.210	3.827
Stress level	0.014 (0.068)	0.833	0.887	1.015	1.169
Resilience	0.562 (0.960)	0.558	0.267	1.754	11.513
Learner’s dimension domain	−0.370 (0.092)	0.000 *	0.577	0.691	0.827
Instructor’s characteristics domain	0.287 (0.135)	0.002 *	1.114	1.333	1.594
Technological characteristics domain	0.163 (0.261)	0.228	0.903	1.177	1.535
Course management and coordination domain	−0.645 (0.195)	0.001 *	0.358	0.525	0.769
Constant	9.703 (4.693)	0.039		16,360.519	

* *p* < 0.05.

## Data Availability

Data supporting results can be requested to the corresponding author who has the dataset archived.
